# Supplementary material for: Shaping micro-clusters via inverse jamming and topographic close-packing of microbombs
Source: Nat Commun. 2017 Sep 28;8:721. doi: 10.1038/s41467-017-00538-z (PMC5620065; doi:10.1038/s41467-017-00538-z)
Supplement: Supplementary file 3 — Supplementary Information [file 41467_2017_538_MOESM3_ESM.pdf]

Description of Supplementary Files

File name: Supplementary Information
Description: Supplementary figures and supplementary table 1

File name: Supplementary Movie 1
Description: Observation of the free-explosion of a microbomb showing the unprecedented volumetric expansion up to 3000%. Note that for the acceleration of the explosion, the solution was heated rapidly (20 °C min^{-1}) from room temperature to 180 °C.

File name: Supplementary Movie 2
Description: Detachment and releasing of the clusters using a dissolvable tape.

File name: Peer Review File

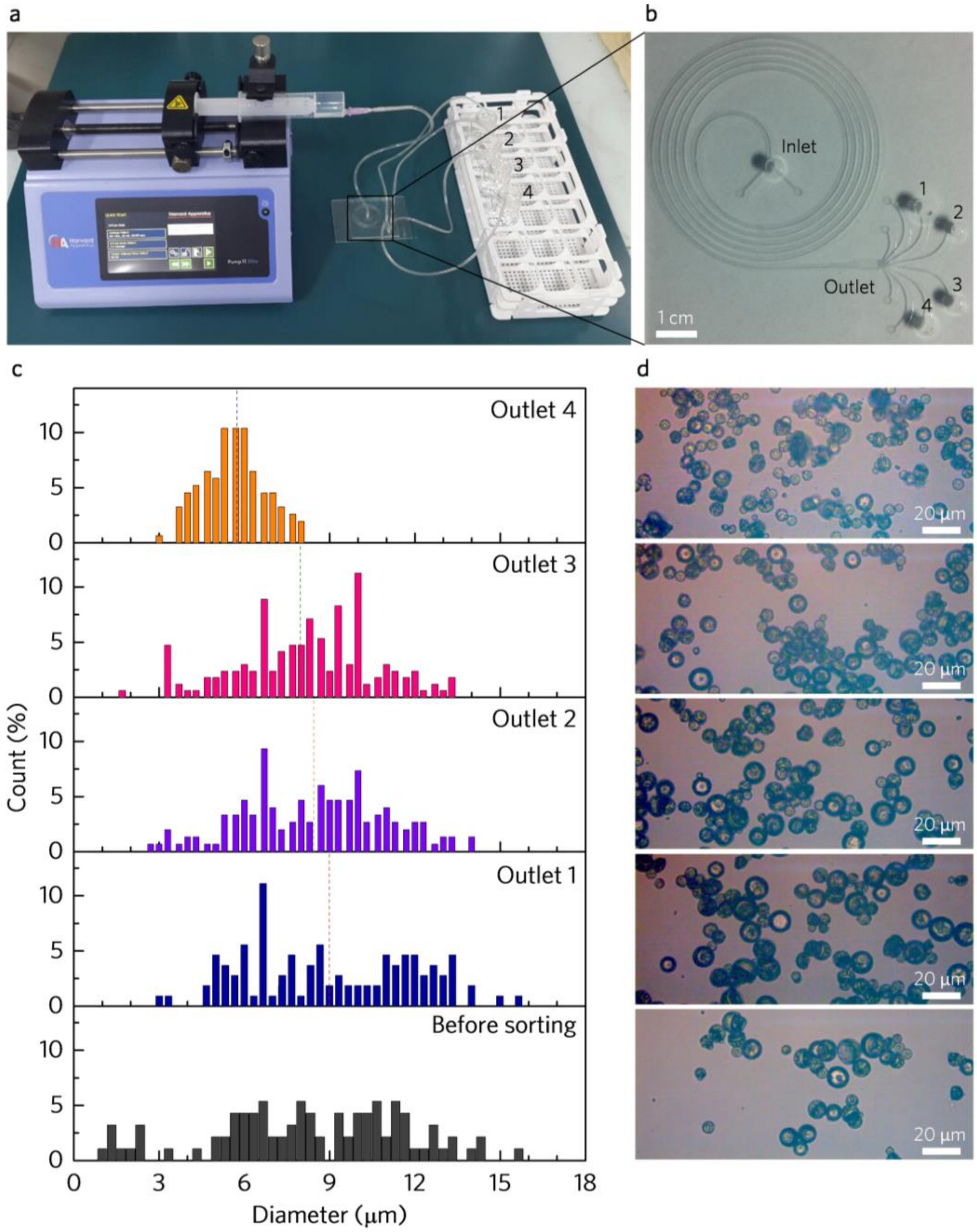

Supplementary Figure 1 | Sorting of microbombs using a spiral microfluidic platform. a, Optical image of the setup with the microfluidic platform for continuous sorting of microbombs that have broad size distribution. The microbombs were separated by inertia and drag force in the platform. **b,** Optical image of the spiral microchannel with one inlet and four outlets. **c,** Statistics of the size distribution of the as-received microbombs and sorted microbombs discharged from four different outlets. **d,** Corresponding optical microscopy images.

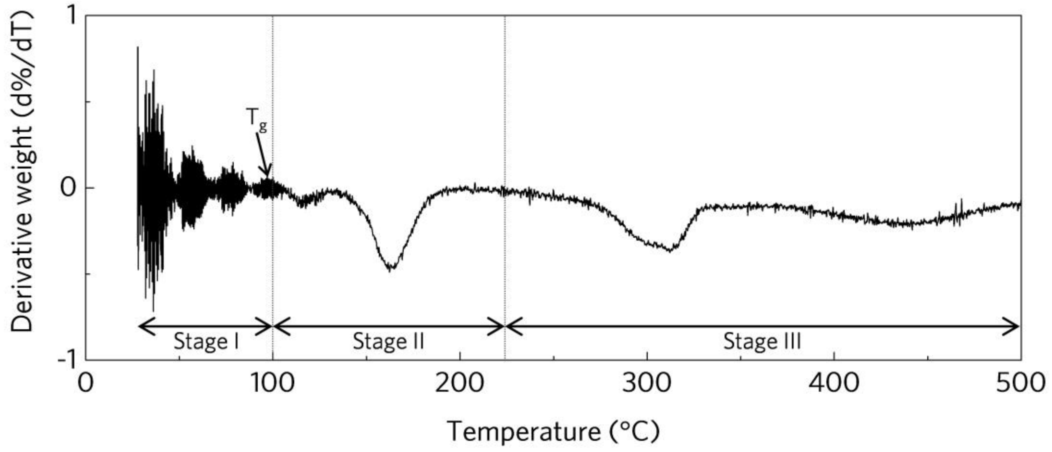

Supplementary Figure 2 | An example of stages of weight loss of microbombs in the case of fast heating (20 °C min^{-1}) by the derivative of weight with respect to the temperature.

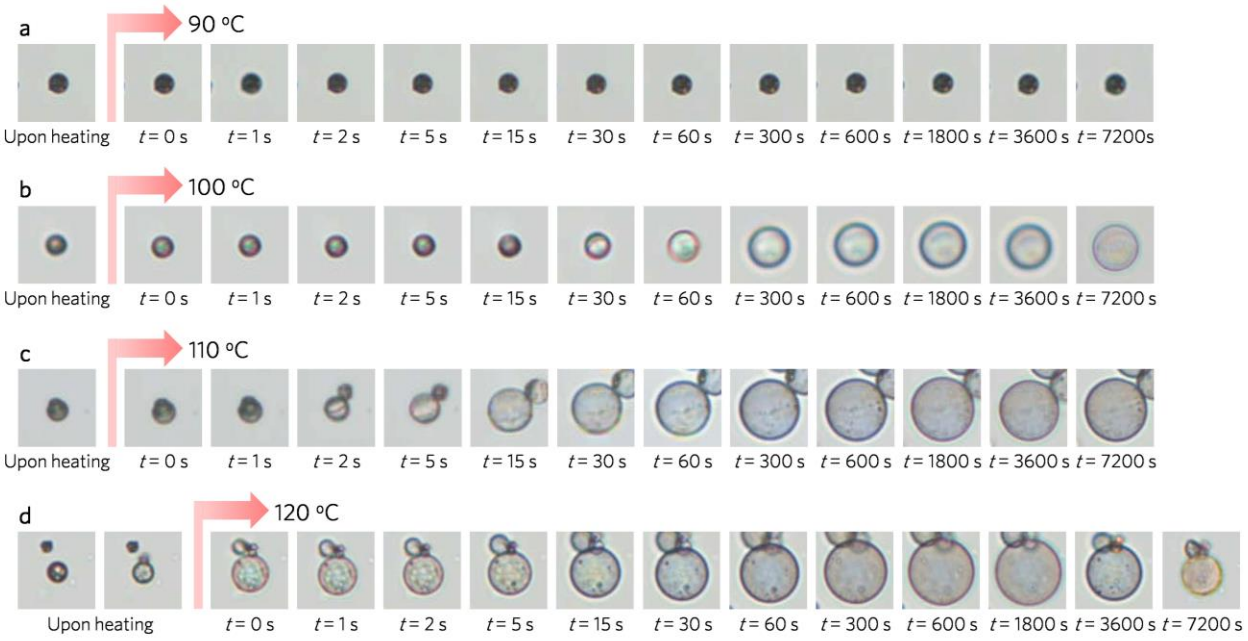

Supplementary Figure 3 | Real-time monitoring of explosions of microbombs. a-d, Isothermal heating at different temperatures: (a) 90, (b) 100, (c) 110, and (d) 120 °C for 2 h to compare the volumetric changes of microbombs over time.

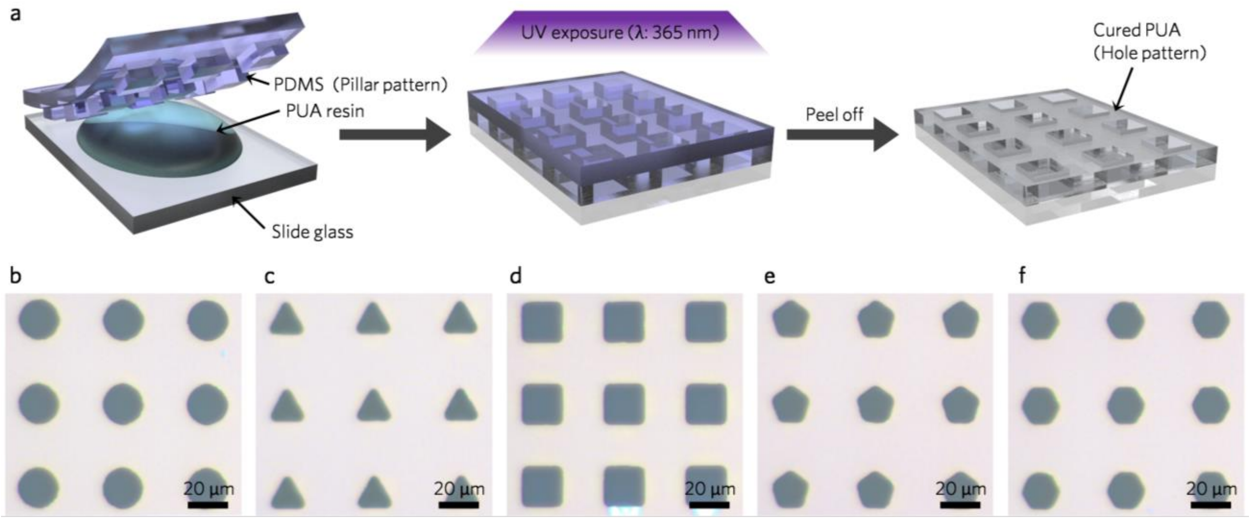

Supplementary Figure 4 | Fabrication of micro-confinement by soft lithography. a, Schematic illustration of the fabrication of microwell structures from PUA resin. **b-f,** Optical microscopy images of the obtained PUA patterns on glass slides with (b) circular, (c) triangular, (d) square, (e) pentagonal, and (f) hexagonal shapes.

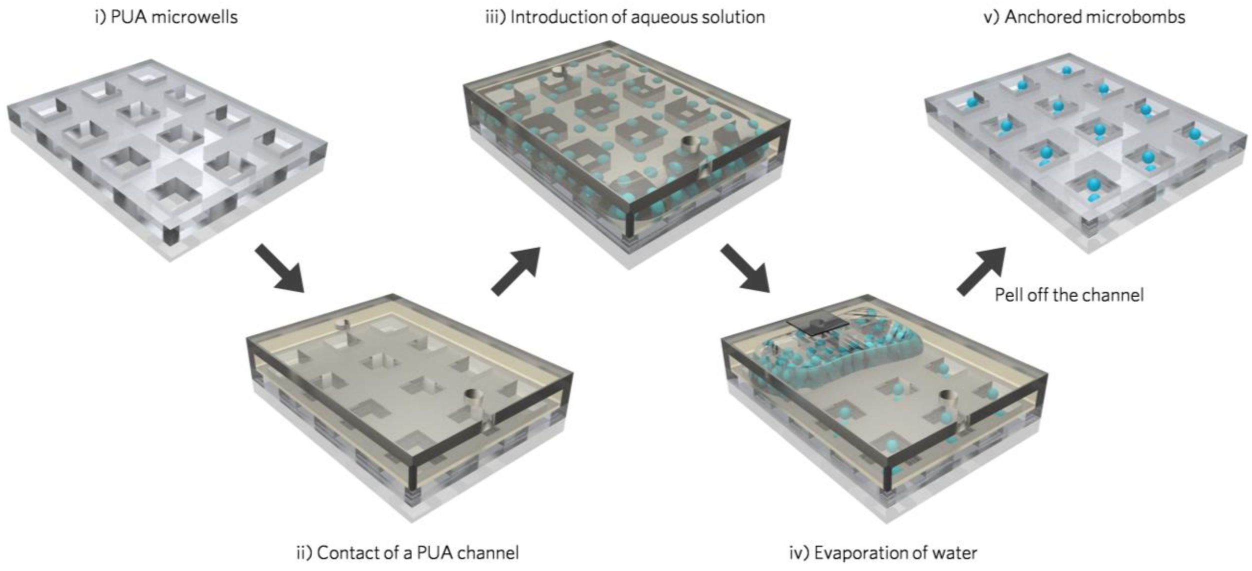

Supplementary Figure 5 | Anchoring the microbombs in the microwells using a temporal microfluidic channel. A PUA channel was positioned to cover the microwells and helped to guide the receding of the meniscus, leading to the anchoring of the microbombs in the microwells. After the anchoring of microbombs, the PUA channel was peeled off from the PUA microwells.

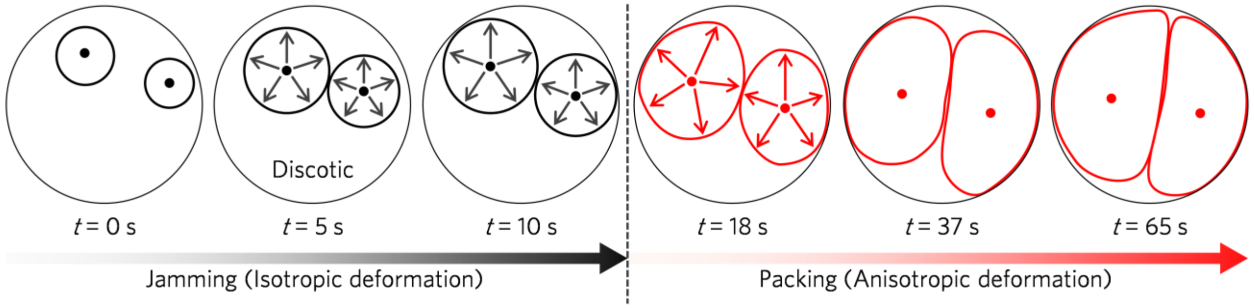

Supplementary Figure 6 | Differentiation of inverse jamming and packing processes during the thermal expansion of the microbombs. The black arrows represent isotropic expansion during the inverse jamming, while the red ones represent asymmetric deformation of boundaries of microbombs yielding topographic close-packing gradually.

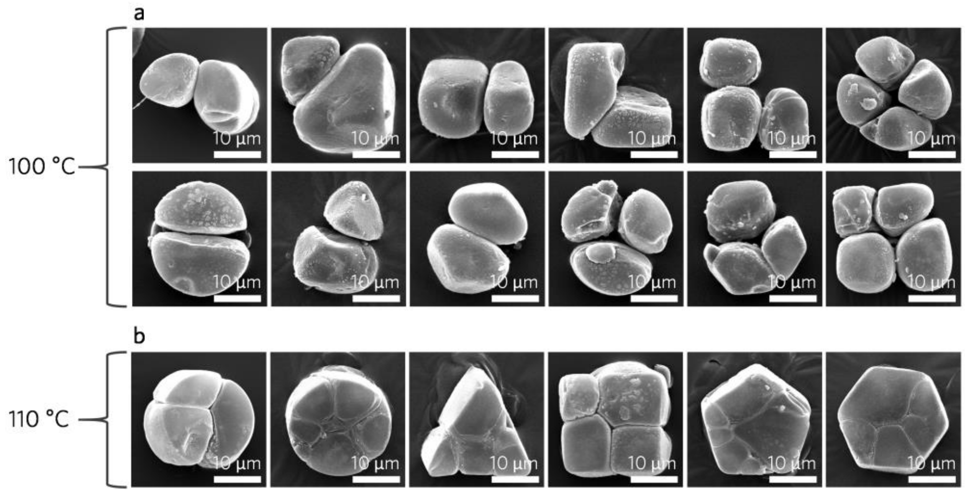

Supplementary Figure 7 | (a, b) SEM images of various clusters with different numbers of cells after heating at 100 and 110 °C for 5 min, respectively, showing clusters with round edges and incomplete connection between cells (**a**) and sharp edges of tight packing with monolithic integration (**b**).

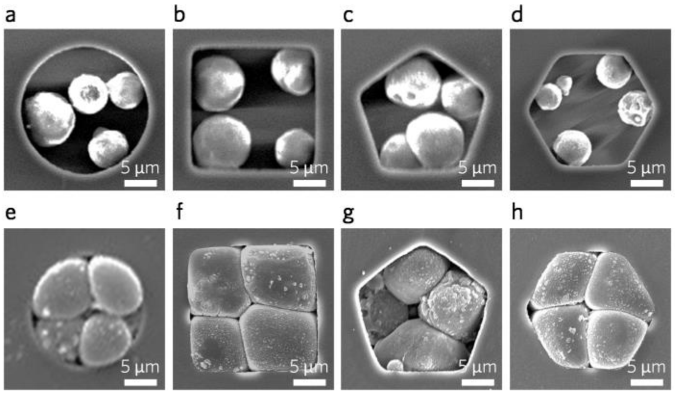

Supplementary Figure 8 | SEM images of clusters with various cleavages formed from four initially separated microbombs in various microwell confinements after heating at 110 °C for 5 min. (a, e) Circular, (b, f) square, (c, g) pentagonal, and (d, h) hexagonal wells.

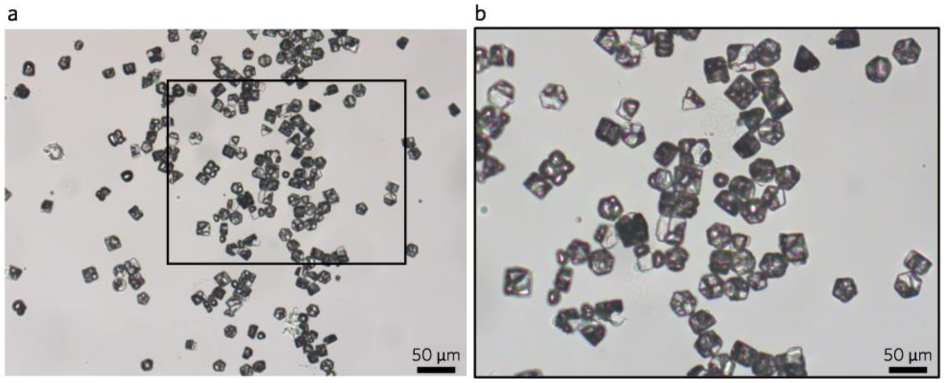

Supplementary Figure 9 | Optical microscopy images of the generated clusters with various shapes.

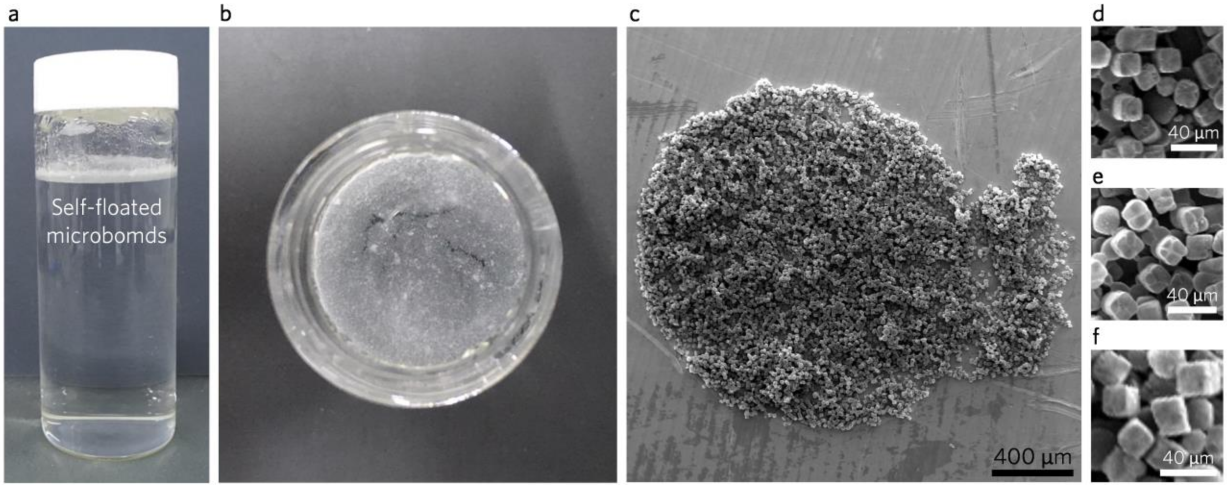

Supplementary Figure 10 | Scalable fabrication of topographic clusters shaped in cubes by exploiting multiple seed expansion. (a, b) Microclusters floating on water after dissolving the water-soluble tape. Note that the hollow nature of our clusters leads to the facile collection of them as shown in the top-view image (b). **(c-f)** SEM images of collected clusters and their morphologies. Note that multiple seed anchoring and expansion enables scalable fabrication of ~ 6.25×10^6 colloidal particles shaped in cubes after a single batch process. The well size is 20 μm × 20 μm × 20 μm with 1:1 spacing ratio from the 10×10 cm^2 patterned area.

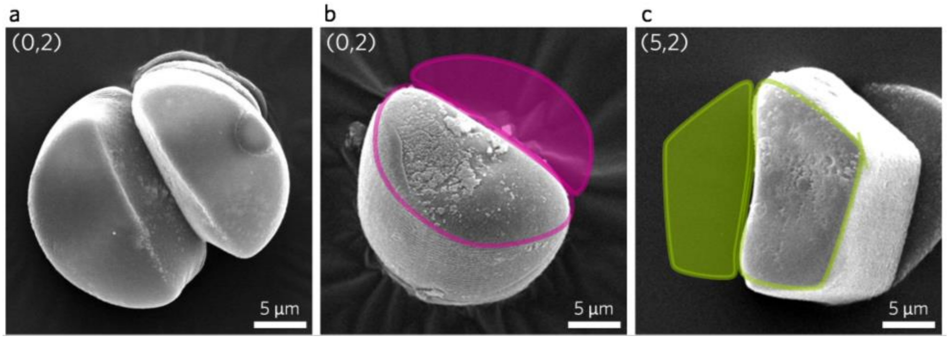

Supplementary Figure 11 | SEM images showing partially disassembled microclusters by reducing the surface-to-surface interaction. Lubricant was applied to the microcapsules prior to expansion to prevent the bonding between seeds.

Supplementary Table 1 | Vapor pressure of isobutane blowing agent as a function of temperature.

**Temperature**	**K**	310	320	330	340	350	360	370	380	390	400	405	407
	**ºC**	36.8	46.8	56.8	66.8	76.8	86.8	96.8	106.8	116.8	126.8	131.8	133.8
**Vapor pressure**	**kPa**	488	633	807	1014	1258	1542	1872	2252	2687	3187	3466	3584
